# Current status and features of antipsychotic prescriptions in Japanese forensic psychiatric wards based on a forensic inpatient database

**DOI:** 10.1002/npr2.12505

**Published:** 2024-11-26

**Authors:** Koji Takeda, Hiroko Kashiwagi, Keisuke Takanobu, Ryotaro Kubota, Ryo Naoe, Yuji Yamada, Junko Koike, Toshiaki Kono, Yuki Kako, Naotsugu Hirabayasi

**Affiliations:** ^1^ Department of Forensic Psychiatry, National Center Hospital National Center of Neurology and Psychiatry Kodaira Tokyo Japan; ^2^ Forensic Psychiatry Center Hokkaido University Hospital Sapporo Hokkaido Japan; ^3^ Department of Community Mental Health and law, National Institute of Mental Health National Center of Neurology and Psychiatry Kodaira Tokyo Japan

**Keywords:** antipsychotic medications, crime, forensic psychiatry, psychopharmacotherapy, schizophrenia spectrum disorders

## Abstract

**Aim:**

Psychopharmacotherapy plays an important role in the treatment of mentally disordered offenders (MDOs) with schizophrenia spectrum disorders. However, there have been few large‐scale reports from multiple forensic psychiatric wards. This study aimed to clarify the current state of antipsychotic medications for MDOs with schizophrenia spectrum disorders in Japanese forensic psychiatric wards.

**Methods:**

Medical information, including age, sex, psychiatric diagnosis, index offense, seclusion or restraint experience during hospitalization, and medication for patients discharged from 32 forensic wards nationwide between September 1, 2019 and December 31, 2021 was provided by the Database Scientific Utilization Project of Japanese forensic psychiatric wards. We analyzed the data of MDOs with schizophrenia spectrum disorders who were prescribed psychotropic medications at the time of discharge, especially focusing on comparing differences between the three groups (clozapine, long‐acting injection (LAI), and other medications).

**Results:**

A total of 362 MDOs with schizophrenia spectrum disorders were prescribed psychotropic medications at discharge. The prescription rates of clozapine and LAI were 23.2% and 24.9%, respectively. Additionally, the rate of antipsychotic polypharmacy was 37.8%. Among the three groups, the clozapine group had the highest rate of seclusion experience (46.4%), a long mean length of hospitalization (1758 days), and the lowest rate of antipsychotic polypharmacy (4.8%). Olanzapine was the most commonly prescribed antipsychotic medication.

**Conclusion:**

This study revealed the current state of antipsychotic medications for MDOs admitted to forensic psychiatric wards in Japan. Future studies are needed to clarify the relevance of antipsychotic medications in the prognosis of MDOs.

## Abstract 2

We analyzed the current status of antipsychotics for Japanese forensic inpatients with schizophrenia spectrum disorders nationwide. Olanzapine was the most commonly prescribed antipsychotic, followed by clozapine. Clozapine was associated with longer hospitalization and a higher rate of seclusion experience.

## Graphical abstract

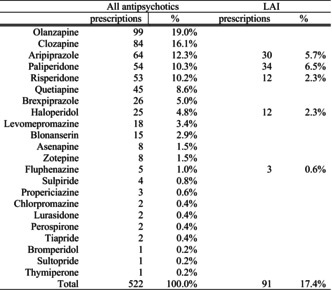

## 1 INTRODUCTION

In Japan, specialized forensic mental health service is provided under the Medical Treatment and Supervision Act (MTSA).^1^ Only those adjudged by the court or prosecutors to be insane or to have diminished responsibility at the time of criminal behavior are subject to the MTSA. Criminal behaviors subject to the MTSA are also limited to homicide, bodily injury, arson, robbery, and sexual assault. The forensic mental health service under the MTSA consists of inpatient and outpatient treatment orders, and approximately 90% of mentally disordered offenders (MDOs) subject to the MTSA initiate treatment under inpatient treatment orders in forensic psychiatric wards. These wards were established at inpatient facilities designated by the Ministry of Health, Labour and Welfare. As of April 1, 2023, there were 856 forensic psychiatric beds in 35 designated inpatient facilities nationwide. The average duration of hospitalization in forensic psychiatric wards is approximately 3 years, with over 80% of patients transitioning to outpatient treatment orders after discharge.^2^ In principle, an outpatient treatment order typically lasts for 3 years, during which psychiatric care and probation are mandatory.

After criminal responsibility judgments by the court or prosecutors, upon the prosecutor's allegation regarding the MTSA, the District Court determines whether to impose treatment orders under the MTSA on MDOs based on a psychiatric evaluation. The key criteria for the MTSA psychiatric evaluation are the “presence of mental disorders,” “treatability,” and “factors impeding the person's reintegration into society.”^3^ “Treatability” refers to the potential response of mental disorders to medical treatments. Consequently, MDOs with schizophrenia spectrum disorders constitute approximately 80% of the patients admitted to forensic psychiatric wards in Japan.^2^ It also might be associated with an elevated risk of violence in patients with schizophrenia.^4^ Therefore, the role of pharmacotherapy, particularly antipsychotic medications, is significant in Japanese forensic psychiatric wards, although the precise situation remains unclear.

Forensic psychiatric patients are complex populations that frequently present with various comorbidities, such as substance‐related and personality disorders.^5^ In addition to improving psychiatric symptoms and preventing relapse, the prescription of antipsychotic drugs for MDOs with schizophrenia spectrum disorders must be considered to reduce the risk of violence. These often lead to differences in prescription practices compared with general psychiatric care.^6^

A study reported a 45% reduction in violence risk during periods of receiving antipsychotic medication compared to periods not on medication.^7^ Among antipsychotics, clozapine reduces violence and aggression in patients with schizophrenia and other psychotics.^8^, ^9^, ^10^ A recent report found that clozapine reduced the risk of violence in patients with schizophrenia with conduct disorders compared to olanzapine and haloperidol.^11^ A systematic review of controlled trials of psychotropic prescriptions in forensic psychiatry included 10 articles.^12^ Of these, five were studies on the effects of clozapine with positive results. However, all the studies had a high risk of bias and a low level of evidence.^12^

A study from 16 countries worldwide from 2005 to 2014 reported that atypical antipsychotic use increased in all populations, and quetiapine, risperidone, and olanzapine were the most frequently prescribed antipsychotics, although the rate and patterns of antipsychotic prescriptions varied markedly between countries.^13^ On the other hand, clozapine is often prescribed to forensic psychiatric patients.^14^, ^15^

Long‐acting injection (LAI) is known to improve adherence to antipsychotic medications.^16^ A Swedish study suggested that LAI may be associated with a reduced risk of violent crimes.^7^ Additionally, a review reported a high prescribing rate of LAI in high security and forensic wards in the United Kingdom, Norway, and Austria, and suggested that LAI might have clinical benefits in patients with schizophrenia exhibiting a high risk of violent behavior.^17^

Although antipsychotic polypharmacy (APP) is not recommended in most treatment guidelines, primarily because of its greater risk of side effects than antipsychotic monotherapy (APM), a recent review suggested that APP may be applicable in cases where non‐clozapine or clozapine APM is ineffective, or clozapine is contraindicated.^18^ A systematic review of the prevalence and correlates of APP (147 studies, 1 418 163 participants, and 82.9% diagnosed with schizophrenia) reported a median APP rate of 19.6%,^19^ and the Asian Psychotropic Prescription 2016 survey reported an APP rate of 42.6% in Asian patients.^20^ Recent studies of antipsychotic prescriptions for forensic psychiatric patients reported high rates of APP.^14^, ^15^, ^21^ In one study, APP was positively correlated with increased length of hospitalization, high/medium security levels, treatment with clozapine, and depot antipsychotic prescriptions.^15^

These perspectives on antipsychotics in general and forensic psychiatric patients have been reported; however, there are few large‐scale multicenter studies on antipsychotic pharmacotherapy for MDOs with schizophrenia spectrum disorders in forensic psychiatric wards. Therefore, this study aimed to reveal the actual state of antipsychotic prescriptions for MDOs on the schizophrenia spectrum disorders in Japanese forensic psychiatric wards using a dataset collected by the Japanese forensic inpatient database.^22^ Additionally, we aimed to identify factors related to antipsychotic prescriptions.

## 2 METHODS

This study was conducted using data collected by the MTSA Database Project.^22^ The MTSA Database Project is a project that collects medical information from Japanese forensic psychiatric wards nationwide. Each facility provides anonymized data to the National Center of Neurology and Psychiatry (NCNP) Hospital, the organizer hospital, via a VPN, which consists of 24 categories broadly classified into *“basic information,” “Orders and hospitalizations under the MTSA,” “treatment process,” “criminal and medical treatment history,” and “problematic behavior in the unit,”* and more than 8000 data items have been collected.^22^As of December 31, 2021, there were 33 designated inpatient facilities in the MTSA, of which 32 institutions sent data to the MTSA Database Project. The organizer hospital provides feedback statistical data to the Ministry of Health, Labor and Welfare and each facility for equalization, generalization, and further improvement of medical care in forensic psychiatric wards. The NCNP has conducted an MTSA database scientific utilization project, and researchers working at designated inpatient facilities of the MTSA can apply to use the data for their research. If the committee of the MTSA database scientific utilization project approves the researcher's application, the data will be re‐anonymized and provided to the extent necessary for the implementation of the research.

### 2.1 Participants and measures

In January 2023, we received data on MDOs who were discharged from 32 designated inpatient facilities of the MTSA between September 1, 2019 and December 31, 2021. The data included age, sex, diagnosis of mental illness classified based on the ICD‐10, index offenses, alcohol and drug problems, duration of hospitalization, behavioral restrictions (seclusion or physical restraint) during hospitalization, year and month of discharge, the presence of outpatient treatment order and residence at discharge, and psychotropic prescriptions at discharge. Data on psychotropic prescriptions included antipsychotics, anxiolytics and hypnotics, antidepressants, mood stabilizers, and antiparkinsonians (Appendix A). Chlorpromazine (CPZ) dose equivalence was also provided. CPZ dose equivalence was calculated using Inada and Inagaki's reports, which were specially designated for Japanese patients, considering racial differences.^23^, ^24^

### 2.2 Procedure

The number of patients who provided data was 468. In this study, patients classified as F2 according to ICD‐10 were defined as patients with schizophrenia spectrum disorder. Thus, of the 468 patients, 88 patients with psychiatric disorders that did not include F2 were excluded from the study. Additionally, 14 patients without psychotropic prescriptions information at discharge were excluded. Finally, four patients who were discharged owing to death were excluded. Alcohol and drug variables were excluded from the analysis because of the large amount of unknown data. Index offenses included attempted offenses. One index offense per one MDO was included in the analysis. MDOs with no antipsychotics were omitted from the analysis of CPZ dose equivalence and Antipsychotic polypharmacy. MDOs who were prescribed brexpiprazole (*n* = 26) or lurasidone (*n* = 2), for which CPZ dose equivalence was not provided by Inada and Inagaki's reports,^23^, ^24^ were excluded from statistics analysis of CPZ dose equivalence.

### 2.3 Statistical analysis

All statistical analyses were performed using IBM SPSS Statistics version 26.0 (IBM Corp).  We defined statistical significance at *p* < 0.05 in this study. Demographic characteristics (age group and sex) were compared between the three groups: Clozapine (patients who were prescribed clozapine at discharge), LAI (patients who were prescribed LAI at discharge), and other medications (patients who were not prescribed clozapine or LAI) using chi‐square tests.

Group differences in the duration of hospitalization and CPZ dose equivalent were examined using a general linear model predicting each variable of interest with groups and sex because there were significant differences in the sex ratio among the three groups. Post‐hoc Bonferroni tests were performed for variables with significant group effects. The Mantel–Haenszel test was used for categorical data, and sex was added to the layer to control for sex.

### 2.4 Ethics

This study was approved by the ethics committees of NCNP (A2022‐025) and Hokkaido University (023‐0080). Approval was obtained from the Committee of the MTSA Database Scientific Utilization Project (MTSA‐006). We conducted this study in accordance with the Declaration of Helsinki. We also complied with the Ethical Guidelines for Medical and Health Research Involving Human Participants, which were established by the Ministry of Education, Culture, Sports, Science, and Technology, the Ministry of Health, Labour and Welfare, and the Ministry of Economy, Trade, and Industry. Written informed consent was waived because this study was conducted using anonymized existing data. Participants were provided with the opportunity to opt out by posting the study information on the NCNP website.

## 3 RESULTS

### 3.1 Demographic information

Table 1 shows the demographics of participants. There was a significant difference in the sex ratio among the three groups (*p* = 0.015); males were more common in the LAI group than in the other medications group (*p* < 0.001). The number of MDOs with F2 (schizophrenia, schizotypal, and delusional disorders) who were prescribed antipsychotics at discharge was 362. Of these, 84 (23.2%) patients were prescribed clozapine, 90 (24.9%) were prescribed LAI, and 188 (51.9%) were not prescribed clozapine or LAI. None of the patients were prescribed both clozapine and LAI. Most patients in each group were in their 30s–50s. F7 (mental retardation) and F8 (disorders of psychological development) were the most common psychiatric comorbidities (7.5%). Bodily injuries (39.0%), homicide (34.3%), and arson (18.5%) were the most commonly reported index offenses.

**TABLE 1:** Demographic information.

Variables	Total	Clozapine	LAI	Other medications	*F*‐statistics *X* ^2^‐statistics	Post hoc Bonferroni test/Mantel–Haenszel test		
						CLZ vs. LAI	CLZ vs. other medications	LAI vs. other medications
	*N* = 362	*N* = 84 (23.2%)	*N* = 90 (24.9%)	*N* = 188 (51.9%)				
Ages (years)								
20–29	30 (8.3%)	7 (8.3%)	6 (6.7%)	17 (9.0%)	*X* ^2^ = 10.994 *p* = 0.529			
30–39	80 (22.1%)	18 (21.4%)	19 (21.1%)	43 (22.9%)				
40–49	110 (30.4%)	29 (34.5%)	28 (31.1%)	53 (28.2%)				
50–59	71 (19.6%)	21 (25.0%)	17 (18.9%)	33 (17.6%)				
60–69	46 (12.7%)	8 (9.5%)	14 (15.6%)	24 (12.8%)				
70–79	23 (6.4%)	1 (1.2%)	6 (6.7%)	16 (8.5%)				
80–89	2 (0.6%)	0 (0%)	0 (0%)	2 (1.1%)				
Sex (males/females)	269 (74.3%) /93 (25.7%)	66 (78.6%)/18 (21.4%)	75 (83.3%)/15 (16.7%)	128 (68.1%)/60 (31.9%)	***X*** ^**2**^ **= 8.453** ***p* = 0.015**	*X* ^2^ = 0.641 *p* = 0.423	*X* ^2^ = 3.121 *p* = 0.077	***X*** ^**2**^ **= 7.183** ***p* < 0.001**
Psychiatric diagnosis (F20–29)								
Schizophrenia	318 (87.8%)	81 (95.2%)	86 (95.6%)	151 (80.3%)				
Schizoaffective disorders	16 (4.4%)	2 (2.4%)	2 (2.2%)	12 (6.4%)				
Delusional disorders	25 (6.9%)	1 (1.2%)	2 (2.2%)	22 (11.7%)				
Acute and transient psychotic disorders	2 (0.6%)	0 (0.0%)	0 (0.0%)	2 (1.1%)				
Schizotypal disorder	1 (0.3%)	0 (0.0%)	0 (0.0%)	1 (0.5%)				
Psychiatric comorbidity								
F00–09	3 (0.8%)	1 (1.2%)	1 (1.1%)	1 (0.5%)				
F10–19	7 (1.9%)	1 (1.2%)	2 (2.2%)	4 (2.1%)				
F60–69	1 (0.3%)	1 (1.2%)	0 (0.0%)	0 (0.0%)				
F70–79	27 (7.5%)	10 (11.9%)	6 (6.7%)	11 (5.9%)				
F80–89	27 (7.5%)	8 (9.5%)	4 (4.4%)	15 (8.0%)				
F90–98	4 (1.1%)	1 (1.2%)	0 (0.0%)	3 (1.6%)				
Index offense								
Homicide	124 (34.3%)	28 (33.3%)	36 (40.0%)	60 (31.9%)	*X* ^2^ = 6.949 *p* = 0.542			
Bodily injury	141 (39.0%)	34 (40.5%)	36 (40.0%)	71 (37.8%)				
Arson	67 (18.5%)	14 (16.7%)	11 (12.2%)	42 (22.3%)				
Robbery	15 (4.1%)	5 (6.0%)	2 (2.2%)	8 (4.3%)				
Sexual assaults	15 (4.1%)	3 (3.6%)	5 (5.6%)	7 (3.7%)				

*Note*: Diagnostic categories are based on ICD‐10 codes as follows: F00–09 (organic, including symptomatic and mental disorders); F10–19 (mental and behavioral disorders due to psychoactive substance use); F20–29 (schizophrenia, schizotypal, and delusional disorders); F60–69 (disorders of adult personality and behavior); F70–79 (mental retardation); F80–89 (disorders of psychological development); F90–98 (behavioral and emotional disorders with onset usually occurring in childhood and adolescence). Bold values denote statistical significance at *p* < 0.05.

Index offense: One index offense per one MDO was included in the analysis. If more than one index offense is recognized per patient, the index offense is selected in the following order. Homicide, arson, robbery, bodily injury, and sex offense.

Abbreviation: LAI, long‐acting injection.

### 3.2 Variables of interest for total, clozapine, LAI, and other medication groups among MDOs with schizophrenia spectrum disorders

Table 2 shows the duration of hospitalization, experience of restrictions, residence at the time of discharge, CPZ dose equivalence, and concomitant rates of other psychotropic prescriptions. The mean duration of hospitalization for all participants was 1240 days. The mean length of hospitalization in the clozapine group was 1758 days, which was significantly longer than that in the LAI (1108 days) and other medications (1071 days) groups (clozapine vs. LAI, *p* < 0.001; clozapine vs. other medications, *p* < 0.001). Overall, 76 patients (21.0%) experienced seclusion during their hospitalization in forensic psychiatric wards, with the clozapine group having a significantly higher rate of seclusion experience (46.4%) than the LAI (13.3%) and other medications (13.3%) groups (clozapine vs. LAI, *p* < 0.001; clozapine vs. other medications, *p* < 0.001).

**TABLE 2:** Variables of interest for total, clozapine, LAI, and others among patients with schizophrenia spectrum disorders.

Variables	Total (*N* = 362)			Clozapine (*N* = 84)			LAI (*N* = 90)			Other medications (*N* = 188)			*F*‐statistics *X* ^2^‐statistics	Post hoc Bonferroni test/Mantel–Haenszel test		
	*N*	Mean	SD	*N*	Mean	SD	*N*	Mean	SD	*N*	Mean	SD		CLZ vs. LAI	CLZ vs. other medications	LAI vs. other medications
Duration of hospitalization (days)	362	1240	683	84	1758	893	90	1108	555	188	1071	493	***F* = 37.20** ***p* < 0.001**	***p* < 0.001**	***p* < 0.001**	*p* = 1.00
Experience of restriction during hospitalization in forensic psychiatric wards																
Seclusion experience	76 (21.0%)			39 (46.4%)			12 (13.3%)			25 (13.3%)			***X*** ^**2**^ **= 42.659** ***p* < 0.001**	***X*** ^**2**^ **= 21.402** ***p* < 0.001**	***X*** ^**2**^ **= 31.251** ***p* < 0.001**	*X* ^2^ = 0.000 *p* = 0.995
Physical restraint experience	22 (6.1%)			9 (10.7%)			4 (4.4%)			9 (4.8%)			*X* ^2^ = 4.133 *p* = 0.127			
Post‐discharge																
End of treatment under the MTSA	44 (12.2%)			13 (15.5%)			3 (3.3%)			28 (14.9%)			***X*** ^**2**^ **= 8.748** ***p* = 0.013**	***X*** ^**2**^ **= 6.052** ***p* = 0.014**	*X* ^2^ = 0.013 *p* = 0.910	***X*** ^**2**^ **= 5.670** ***p* = 0.017**
Transferred to the outpatient treatment order under the MTSA																
Hospitalization to general psychiatric wards	48 (13.3%)			15 (17.9%)			13 (14.4%)			20 (10.6%)			*X* ^2^ = 2.777 *p* = 0.249			
Welfare facilities	151 (41.7%)			37 (44.0%)			34 (37.8%)			80 (42.6%)			*X* ^2^ = 0.816 *p* = 0.665			
Living with family	49 (13.5%)			7 (8.3%)			16 (17.8%)			26 (13.8%)			*X* ^2^ = 3.340 *p* = 0.188			
Living alone	70 (19.3%)			12 (14.3%)			24 (26.7%)			34 (18.1%)			*X* ^2^ = 4.663 *p* = 0.097			
Medication (at the time of discharge from forensic psychiatric wards)																
CPZ dose eq. (mg/day)	329	759	560	83	766	684	88	786	498	158	741	521	*F* = 0.006 *p* = 0.994			
Antipsychotic polypharmacy	135 (37.8%)			4 (4.8%)			52 (57.8%)			79 (43.2%)			***X*** ^**2**^ **= 56.509** ***p* < 0.001**	***X*** ^**2**^ **= 52.534** ***p* < 0.001**	***X*** ^**2**^ **= 39.814** ***p* < 0.001**	*X* ^2^ = 3.168 *p* = 0.075
Antiparkinsonians	80 (22.1%)			15 (17.9%)			25 (27.8%)			40 (21.3%)			*X* ^2^ = 2.638 *p* = 0.267			
Antidepressants	24 (6.6%)			7 (8.3%)			6 (6.7%)			11 (5.6%)			*X* ^2^ = 0.578 *p* = 0.749			
Mood stabilizers	123 (34.0%)			52 (61.9%)			18 (20.0%)			53 (28.2%)			***X*** ^**2**^ **= 39.848** ***p* < 0.001**	***X*** ^**2**^ **= 29.133** ***p* < 0.001**	***X*** ^**2**^ **= 26.206** ***p* < 0.001**	*X* ^2^ = 1.793 *p* = 0.181
Anxiolytics and hypnotics	196 (54.1%)			44 (52.4%)			42 (46.7%)			110 (58.5%)			*X* ^2^ = 3.576 *p* = 0.167			

*Note*: Participants with no antipsychotics were omitted from the analysis of CPZ dose eq. and antipsychotic polypharmacy. Participants who were prescribed drugs of which chlorpromazine equivalents could not be calculated were omitted from the analysis of CPZ dose eq. Bold values denote statistical significance at *p* < 0.05.

Abbreviations: CPZ, chlorpromazine; eq, equivalent; LAI, long‐acting injection; MTSA, Medical Treatment and Supervision Act.

The proportion of those who completed the MTSA treatment order at the time of discharge from the forensic psychiatric ward was 12.2%, with a significantly smaller proportion in the LAI group (3.3%) than in the clozapine and other medications groups (LAI vs. clozapine, *p* = 0.014; LAI vs. other medications, *p* = 0.017). The most common residential status of participants who received an outpatient treatment order after discharge from forensic psychiatric wards was welfare facilities (41.7%), followed by living alone (19.3%), living with family (13.5%), and hospitalization in general psychiatric wards (13.3%). The mean CPZ dose equivalent of antipsychotic prescriptions was 759 mg/day, with no significant differences among the groups. The overall rate of APP was 37.8%. The rate of APP in the clozapine group (4.8%) was significantly lower than that in the LAI (57.8%) and other medications (43.2%) groups (clozapine vs. LAI, *p* < 0.001; clozapine vs. other medications, *p* < 0.001). The concomitant prescription rates of antiparkinsonians, antidepressants, mood stabilizers, and anxiolytics/hypnotics were 22.1%, 6.6%, 34.0%, and 54.1%, respectively. The rate of concomitant prescription of mood stabilizers in the clozapine group was 61.9%, which was significantly higher than that in the LAI (20.0%) and other medications (28.2%) groups (clozapine vs. LAI, *p* < 0.001; clozapine vs. other medications, *p* < 0.001).

### 3.3 Prescription details of antipsychotic medications and mood stabilizers

Table 3 shows the status of antipsychotic prescriptions for MDOs with schizophrenia spectrum disorders at discharge (all medication routes were combined). The proportion of antipsychotic medication was calculated as the number of prescriptions for each antipsychotic/the number of prescriptions for all antipsychotics. Olanzapine was the most commonly prescribed antipsychotic (19.0%), followed by clozapine (16.1%), aripiprazole (12.3%), paliperidone (10.3%), risperidone (10.2%), and quetiapine (8.6%). When limited to LAI, paliperidone (6.5%) and aripiprazole (5.7%) were prescribed at higher rates.

**TABLE 3:** Antipsychotic medications.

	All antipsychotics		LAI	
	Prescriptions	%	Prescriptions	%
Olanzapine	99	19.0%		
Clozapine	84	16.1%		
Aripiprazole	64	12.3%	30	5.7%
Paliperidone	54	10.3%	34	6.5%
Risperidone	53	10.2%	12	2.3%
Quetiapine	45	8.6%		
Brexpiprazole	26	5.0%		
Haloperidol	25	4.8%	12	2.3%
Levomepromazine	18	3.4%		
Blonanserin	15	2.9%		
Asenapine	8	1.5%		
Zotepine	8	1.5%		
Fluphenazine	5	1.0%	3	0.6%
Sulpiride	4	0.8%		
Propericiazine	3	0.6%		
Chlorpromazine	2	0.4%		
Lurasidone	2	0.4%		
Perospirone	2	0.4%		
Tiapride	2	0.4%		
Bromperidol	1	0.2%		
Sultopride	1	0.2%		
Thymiperone	1	0.2%		
Total	522	100.0%	91	17.4%

*Note*: All antipsychotics include oral and LAI antipsychotic prescriptions.

Abbreviation: LAI, long‐acting injection.

Valproic acid (65 patients; 18.0%) was the most commonly prescribed mood stabilizers, followed by lithium (64 patients; 17.7%). In the clozapine group, 47.6% of patients were prescribed lithium and 21.4% were prescribed VPA (Table 4).

**TABLE 4:** Concomitant rate of mood stabilizers.

	Total (*N* = 362)		Clozapine (*N* = 84)		LAI (*N* = 90)		Other medications (*N* = 188)	
	*N*	%	*N*	%	*N*	%	*N*	%
Valproic acid	65	18.0%	18	21.4%	12	13.3%	35	18.6%
Lithium	64	17.7%	40	47.6%	6	6.7%	18	9.6%
Lamotrigine	9	2.5%	5	6.0%	0	0.0%	4	2.1%
Carbamazepine	4	1.1%	1	1.2%	0	0.0%	3	1.6%

Abbreviation: LAI, long‐acting injection.

## 4 DISCUSSION

This study examined antipsychotic prescriptions at the time of discharge from forensic psychiatric wards for MDOs with schizophrenia spectrum disorders under the MTSA treatment orders in Japan. To the best of our knowledge, this study is significant because there have been few large‐scale multicenter reports on the status of antipsychotic prescriptions for MDOs with schizophrenia spectrum disorders in forensic psychiatric wards.

Clozapine was prescribed to 23.2% of patients and also accounted for 16.1% of all antipsychotic prescriptions. The proportion of clozapine prescriptions in Japanese forensic psychiatric wards was clearly higher than in Japanese previous reports for general outpatients with schizophrenia.^25^ Japanese forensic psychiatrists may actively prescribe clozapine for MDOs with schizophrenia spectrum disorders owing to the evidence that clozapine reduces violence and aggression in patients with schizophrenia.^8^, ^9^, ^10^

LAI was prescribed to 24.9% of participants and also accounted for 17.4% of all antipsychotic prescriptions. The prescription rate of LAI was also higher than that reported for general outpatients with schizophrenia in Japan.^25^ A review suggested that LAI might be effective in reducing the risk of violence in patients with schizophrenia.^17^ A previous study reported that 41.2% of MDOs under the MTSA treatment orders discontinued psychiatric treatment at the time of their index offenses.^26^ It can be assumed that Japanese forensic psychiatrists consider LAI to be one of the best ways to prevent relapse and reoffending and continue treatment for MDOs with outpatient treatment orders.

In this study, men were more common in the LAI group than in the other medications group, which may be because help‐seeking behavior is more common in women with schizophrenia than men and women are thought to adhere better to treatment.^27^

The rate of seclusion experienced during hospitalization in the forensic psychiatric wards was significantly high in the clozapine group (46.4%). The temporal relationship between clozapine prescription and seclusion experience remains unknown because only psychotropic prescription information at discharge was available for this study; nonetheless, it is assumed that clozapine is prescribed to patients who are sufficiently difficult to require seclusion based on our clinical experience. The clozapine group had the longest length of hospitalization in this study, which can be attributed to two main reasons. First, it was assumed that many of the patients were highly difficult to treat, which is the same reason for the high rate of seclusion experience. Second, the designated outpatient facilities that could prescribe clozapine were limited. Therefore, it might take time to create the community living environment necessary for patients to be discharged from the forensic psychiatric wards.

The overall rate of APP was 37.8%. This was somewhat lower than the APP rate in patients in forensic settings, which was recently reported in Greenland,^14^ Italy,^21^ and Canada,^15^ although it was higher than previous reports from Australia^28^ and the United Kingdom.^29^ In this study, the rate of APP was extremely low (4.8%) in the clozapine group, and no patients were prescribed both clozapine and LAI. In Japan, the low rate of APP was also reported in general patients with schizophrenia who were prescribed clozapine.^30^ This is because, in Japan, regulations require that clozapine be prescribed as monotherapy in principle, and the combination of clozapine and LAI is prohibited. The LAI group tended to have a higher rate of APP than the other medications group (*p* = 0.075), which is consistent with a nationwide Japanese survey of prescriptions at discharge from general psychiatric wards.^31^ The report speculated that Japanese psychiatrists might have judged that the patients with LAI had severe symptoms and prescribed oral antipsychotics in combination with LAI to prevent relapse after discharge.^31^

Among the prescribed antipsychotics, olanzapine and clozapine were the most commonly prescribed. These antipsychotics are also well‐prescribed in forensic settings overseas.^14^, ^15^ The high rankings of aripiprazole, paliperidone, and risperidone might be partly because these drugs can be administered via LAI in Japan (Appendix A).

The rate of concomitant prescriptions of mood stabilizers was significantly high in the clozapine group (61.9%), with a high rate of lithium prescription (47.6%). A systematic review reported the potential benefit of prescribing lithium for clozapine‐related neutropenia,^32^ and it is assumed that Japanese forensic psychiatrists may also have prescribed lithium frequently in anticipation of such a possibility.

The concomitant use of clozapine and sodium valproate can increase the risk of adverse events such as clozapine induced fever,^33^ myocarditis,^34^ and neutropenia.^35^ Although this risk exists, concomitant use of clozapine and sodium valproate is not uncommon in Japan, reported to be 9.6% in a nationwide study.^30^ In this study, the concomitant use rate of sodium valproate for patients with clozapine was even higher, at 21.4%. Japanese forensic psychiatrists should work to reduce the concomitant use of sodium valproate in patients receiving clozapine.

### 4.1 Study limitations

This study has certain limitations. First, we did not obtain information on psychiatric symptom severity, the timing of onset of mental illness, and adverse events caused by psychotropic medications. Therefore, the relationship between those items and antipsychotic prescription status remains unknown.

There are also limitations to the prescription data. The MTSA database comprises an automated extraction system from the electronic medical records of each facility. Prescription data might not be correctly submitted to the database from each facility if it was not correctly recorded in their electronic medical records or if appropriate procedures were not taken when extracting and submitting the data to the database system in each facility.

Moreover, prescription data covered drugs marketed in Japan. Blonanserin tape was launched in September 2019, and the paliperidone palmitate 3 months formulation was launched in December 2020. However, this study did not include information on these drugs because the database system had not collected data for these drugs as of December 31, 2021.

### 4.2 Further prospectives

Although this study clarified the antipsychotic prescribing at discharge, the relationship of antipsychotic prescription with relapse, reoffences, mortality, suicide, and re‐hospitalization after discharge from forensic psychiatric wards remain unknown and are future research challenges. Additionally, no public database in Japan collects data on patients with outpatient treatment orders under the MTSA. Although inpatient care is important in forensic psychiatry, it is even more important to stabilize patients' psychiatric symptoms, improve their quality of life, and prevent reoffences, in their community after discharge. Therefore, a public database of the patients receiving outpatient treatment orders is required.

### 4.3 Conclusion

In this study, we presented the status of antipsychotic prescriptions for MDOs with schizophrenia spectrum disorders at discharge from forensic psychiatric wards in Japan. Olanzapine was the most commonly prescribed antipsychotic, followed by clozapine. Clozapine was associated with longer hospitalization and a higher rate of seclusion experience. Further studies are needed to clarify the association between antipsychotic prescriptions and patient prognosis after discharge from forensic psychiatric wards.

## AUTHOR CONTRIBUTIONS

K. Takeda and H.K. conceived and designed the study and contributed equally. H.K. performed data and statistical analyses. All the authors contributed to drafting the manuscript.

## FUNDING INFORMATION

This study was supported by a Health Labour Sciences Research Grant from the Ministry of Health, Labour and Welfare (202218020A).

## CONFLICT OF INTEREST STATEMENT

K. Takeda received Novartis Research Grant (2019) from Novartis Pharma. H.K., R.N., and R.K. received personal fees from Sumitomo Pharma. Y.Y. received personal fees from Sumitomo Pharma and Lundbeck Japan. Y.K. received personal fees from Otsuka Pharmaceutical, Janssen Pharmaceutical, Yoshitomiyakuhin, Viatris, and Sumitomo Pharma. N.H. received personal fees from Janssen Pharmaceutical, Meiji Seika Pharma, Johnson & Johnson Innovative Medicine, Sumitomo Pharma, Takeda Pharmaceutical, and Otsuka Pharmaceutical. K. Takanobu, J.K., and T.K. have no conflicts of interest to declare.

## ETHICS STATEMENT

Approval of the research protocol by an Institutional Reviewer Board: This study was approved by the ethics committees of NCNP (A2022‐025) and Hokkaido University (023‐0080). Approval was obtained from the Committee of the MTSA Database Scientific Utilization Project (MTSA‐006).

Informed Consent: Written informed consent was waived because this study was conducted using anonymized existing data. Participants were provided with the opportunity to opt out by posting the study information on the NCNP website.

Registry and the Registration No. of the study/trial: n/a.

## Data Availability

Because the data provided by the committee of the MTSA database scientific utilization project includes not only medical information but also records of serious offending, the use of the data must be strictly controlled from the standpoint of privacy protection. According to the terms of the MTSA database scientific utilization project, only those who work in Japanese forensic psychiatric wards can apply to utilize data. The terms also prohibit researchers who receive the data from disclosing, lending, transferring, selling, or providing the data to anyone other than collaborating researchers. Therefore, the data used in this study cannot be shared publicly, and thus the supporting data are not available.

## APPENDIX A List of psychotropic medications in this study

### A.1

**Table:**

Type of drugs	Medication name	LAI
Antipsychotics	Asenapine	
	Aripiprazole	〇
	Oxypertin	
	Olanzapine	
	Carpipramine	
	Quetiapine	
	Clocapramine	
	Clozapine	
	Chlorpromazine	
	Spiperone	
	Sultopride	
	Sulpiride	
	Zotepine	
	Tiapride	
	Thymiperone	
	Trifluoperazine	
	Nemonapride	
	Paliperidone	〇
	Haloperidol	〇
	Pipamperone	
	Pimozide	
	Fluphenazine	〇
	Brexpiprazole	
	Prochlorperazine	
	Blonanserin	
	Propeliciazine	
	Bromperidol	
	Perphenazine	
	Perospirone	
	Mosapramine	
	Moperon	
	Risperidone	〇
	Lurasidone	
	Reserpine	
	Levomepromazine	
Antidepressants	Amitriptyline	
	Amoxapine	
	Imipramine	
	Escitalopram	
	Clomipramine	
	Setiptiline	
	Sertraline	
	Duloxetine	
	Dosulepin	

	Trazodone	
	Trimipramine	
	Nortriptyline	
	Paroxetine	
	Fluvoxamine	
	Venlafaxine	
	Vortioxetine	
	Maprotiline	
	Mianserin	
	Mirtazapine	
	Milnacipran	
Lofepramine		
Mood stabilizers	Carbamazepine	
	Valproic acid	
	Lamotrigine	
	Lithium	
Anxiolytics and hypnotics	Amobarbital	
	Alprazolam	
	Eszopiclone	
	Estazolam	
	Etizolam	
	Oxazolam	
	Quazepam	
	Cloxazolam	
	Clothiazepam	
	Clonazepam	
	Clobazam	
	Clorazepate	
	Chlordiazepoxide	
	Diazepam	
	Zopiclone	
	Zolpidem	
	Tandospirone	
	Tofisopam	
	Triazolam	
	Nitrazepam	
	Nimetazepam	
	Barbital	
	Haloxazolam	
	Phenobarbital	
	Fludiazepam	
	Flutazolam	

	Flunitrazepam	
	Flurazepam	
	Brotizolam	
	Bromazepam	
	Bromovalerylurea	
	Pentobarbital calcium	
	Mexazolam	
	Medazepam	
	Hydrochloride hydrate	
	Ethyl loflazepate	
	Lorazepam	
Lormetazepam		
Antiparkinsonians	Amantadine	
	Diphenhydramine	
	Trihexyphenidyl	
	Hydroxyzine	
	Biperiden	
	Piroheptine	
	Profenamine	
	Promethazine	
	Mazaticol	
	Metixene	

Abbreviations: LAI, long‐acting injection.
